# Adherens, tight, and gap junctions in ependymal cells: A systematic review of their contribution to CSF-brain barrier

**DOI:** 10.3389/fneur.2023.1092205

**Published:** 2023-03-24

**Authors:** Riccardo Serra, J. Marc Simard

**Affiliations:** ^1^Department of Neurosurgery, University of Maryland, Baltimore, MD, United States; ^2^Department of Pathology, University of Maryland, Baltimore, MD, United States; ^3^Department of Physiology, University of Maryland, Baltimore, MD, United States

**Keywords:** ependyma, tight junctions, adherens junctions, gap junctions, hydrocephalus

## Abstract

**Introduction:**

The movement of fluids and solutes across the ependymal barrier, and their changes in physiologic and disease states are poorly understood. This gap in knowledge contributes strongly to treatment failures and complications in various neurological disorders.

**Methods:**

We systematically searched and reviewed original research articles treating ependymal intercellular junctions on PubMed. Reviews, opinion papers, and abstracts were excluded. Research conducted on tissue samples, cell lines, CSF, and animal models was considered.

**Results:**

A total of 45 novel articles treating tight, adherens and gap junctions of the ependyma were included in our review, spanning from 1960 to 2022. The findings of this review point toward a central and not yet fully characterized role of the ependymal lining ultrastructure in fluid flow interactions in the brain. In particular, tight junctions circumferentially line the apical equator of ependymal cells, changing between embryonal and adult life in several rodent models, shaping fluid and solute transit in this location. Further, adherens and gap junctions appear to have a pivotal role in several forms of congenital hydrocephalus.

**Conclusions:**

These findings may provide an opportunity for medical management of CSF disorders, potentially allowing for tuning of CSF secretion and absorption. Beyond hydrocephalus, stroke, trauma, this information has relevance for metabolite clearance and drug delivery, with potential to affect many patients with a variety of neurological disorders. This critical look at intercellular junctions in ependyma and the surrounding interstitial spaces is meant to inspire future research on a central and rather unknown component of the CSF-brain interface.

## 1. Introduction

The ependymal barrier, a simple cuboidal epithelium that lines the ventricular and aqueductal surfaces and interfaces with the brain parenchyma, is one of multiple nodes of intraventricular and extraventricular CSF circulation. For decades the ependyma has been seen as a permeable, metabolically and functionally inactive covering of the cerebral ventricles, aqueduct and central canal of the spinal cord. While the choroid plexus, according to the classic “Third Circulation” theory by Dandy and Blackfan (1, 2), has been considered as the main source of CSF production, recent microscopic and molecular studies have suggested a possible role for perivascular, lymphatic and ependymal pathways in contributing to absorption and secretion of CSF and interstitial fluid. This exchange could be even more important in disease states such as hydrocephalus, Alzheimer's disease, Normal Pressure Hydrocephalus (3), accounting for a larger proportion of fluid production. Further, this new data spurred renewed interest in the field of ependymal paracellular flow and in the changes that intercellular junctions undergo *in utero* and during postnatal development. The transependymal route, while accounting for only about 5–10% of total CSF reabsorption in normal states, may constitute an important route for CSF exchange in pathologic conditions. Junctional proteins such as cadherins, connexins and claudins are at the heart of its barrier function, and alterations in their characteristics, expression, integrity could affect the paracellular flow of solutes and water.

Despite growing evidence pointing toward a pivotal role for ependyma in ventricular development and CSF movement, the full extent of the involvement of intercellular junctions in trans-ependymal flow is still unclear. Further, the physiologic age-related changes in ependymal integrity and permeability have not been fully characterized. Despite recent research suggesting a role in CSF homeostasis as well as primitive neurogenesis, a thorough understanding of ependymal junctional complexes is still missing. Given the recent contributions on the role of these structures in congenital, communicating and obstructive hydrocephalus, the novel techniques used in recent years to assess flows, the discovery of alternative pathways of drainage in the brain and subarachnoid spaces, we sought to review the current state of the evidence on adherens, gap and tight junctions in the ependymal layer. The aim is to provide the reader with the most updated evidence on the role of junctions in fluid, solute flow and development of CNS-related disorders. Starting from a number of foundational studies based on electron microscopy, we integrated that knowledge with information generated with modern fluorescent imaging techniques, to provide a glimpse of the potential role of these proteins in several pathologies of the ependyma and various forms of hydrocephalus.

## 2. Methods

**Figure 1:**
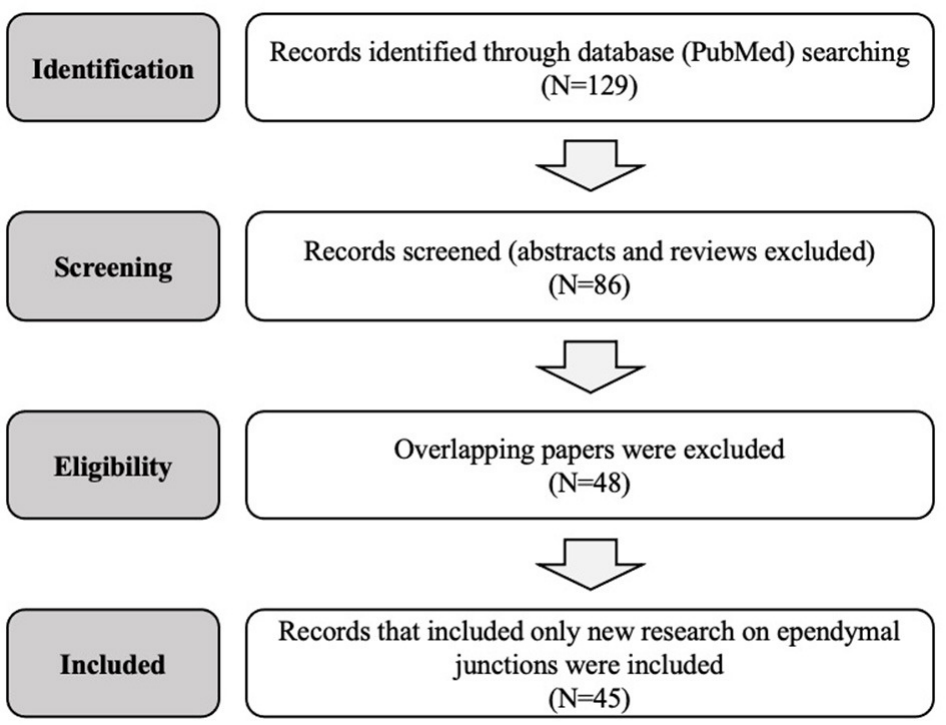
Flow diagram of the systematic research and selection process used to identify the final papers in this review.

This review is limited to original research on the mammalian and non-mammalian ependymal system published in the past 50 years. A search of the PubMed database was conducted for the terms “gap junction,” “tight junction,” “adherens junction,” “ependyma,” as well as specific protein types “claudin,” “occludin,” “JAM,” “zonula,” “cadherin,” and “connexin.” Abstracts were reviewed, only manuscripts presenting new research were selected, and full texts were considered in the final analysis. Abstracts not associated with a published manuscript were excluded. Figure 1 and Table 1 summarize and outline the selection process used for this review. A total of 45 studies that met these criteria were identified and considered for the final manuscript. Data from each of these papers was included in the review and interpreted in the “Discussion” section. This review was conducted according to the PRISMA checklist (41).

**Table 1:** List of the final papers selected for the review.

**Authors**	**Title**	**Journal**	**Methods**	**Findings**
Tennyson et al. (4)	An electron microscope study of ependymal cells of the fetal, early postnatal and adult rabbit.	Zeitschrift für Zellforschung und mikroskopische Anatomie. 1962	Electron microcopy analysis of rabbit ependymal cells.	Simple columnar cell type in the mature rabbit. Ependymal astrocytes and tanycytes are common in embryos. Extensive interdigitations and numerous organelles are present in the latter cellular elements.
Duckett (5)	The germinal layer of the growing human brain during early fetal life.	The Anatomical Record. 1968	Light and electron microscopy of the germinal telencephalon.	The human telencephalon may have an absorptive role between the eighth and fifteenth weeks of fetal life, given its resemblance to the renal epithelium.
Fossan et al. (6)	CSF-brain permeability in the immature sheep fetus: a CSF-brain barrier.	Developmental Brain Research. 1985	Permeability of the neuroependyma between CSF and brain studied in fetal sheep of 60 and 125 days gestation.	Horseradish peroxidase penetration limited to the ventricles in fetuses, but present in the subventricular zone in adults. Lower volumes of distribution of sucrose and insulin in younger animals, with narrower solute distribution.
Møllgård et al. (7)	Cell junctions and membrane specializations in the ventricular zone (germinal matrix) of the developing sheep brain: a CSF-brain barrier.	Journal of neurocytology. 1987	Electron microscopy of neuroependymal cells at day 19–40 of embryonic development.	Continuos tight and gap junctions were identified in the earliest stages, with tight junction spiraling from the ventricular pole toward the deeper ventricular zone. Zonulae adherents, large gap junctions and orthogonal arrays were found in mature ependyma.
Møllgård et al. (7)	The development of the human blood-brain and blood-CSF barriers.	Neuropathology and applied neurobiology. 1986	Horseradish peroxidase and freeze-fracturing in chick, rat and monkey brain.	High CSF protein concentration in the fetal ventricles. Strap junctions were found in the developing germinal matrix.
Brightman and Reese (8)	Junctions between intimately apposed cell membranes in the vertebrate brain.	The Journal of cell biology. 1969	Horseradish peroxidase and lanthanum	Penetration of horseradish peroxidase or lanthanum in the median gap, suggesting the existence of gap junctions and incomplete tight junctions.
Cavanagh and Warren (9)	The distribution of native albumin and foreign albumin injected into lateral ventricles of prenatal and neonatal rat forebrains.	Anatomy and embryology. 1985	Distribution of plasma albumin in the rat forebrain from day 14 of gestation until birth.	Not seen within cells of the developing forebrain until day 16E or 17E. Sheep albumin was taken up rapidly into cells of the ventricular zone at the later but not the earlier ages, suggesting uptake rather than local synthesis.
Dziegielewska et al. (10)	Proteins in cerebrospinal fluid and plasma of fetal rats during development.	Developmental biology. 1981	Total protein, albumin, and α-fetoprotein measured in CSF and plasma of fetal (12 to 22 days gestation) and neonatal (0 to 10 days postnatal) rats	Total protein concentration in plasma increased throughout the developmental period studied as did that of albumin. α-Fetoprotein peaked at day 19 of gestation and then declined. Albumin and α-fetoprotein constituted over 50% of the total protein concentration in csf at all fetal ages.
Dziegielewska et al. (10)	Studies of the development of brain barrier systems to lipid insoluble molecules in fetal sheep.	The Journal of physiology. 1979	Distribution of labeled erythritol (C14), sucrose (3H or 14C), inulin (3H or 14C) and albumin (125I), or albumin and IgG detected by immunoassay in fetal sheep, early (60 days) and late (125 days) in gestation.	60 days: markers seem to penetrate into CSF by diffusion. Reduction in penetration which occurred by 125 days for all markers except erythritol. No change in junctional characteristics (tight junction depth and strand number) between the two ages studied, despite the changes in permeability.
Dziegielewska et al. (10)	Blood-cerebrospinal fluid transfer of plasma proteins during fetal development in the sheep.	The Journal of physiology. 1980	Penetration of human and sheep plasma proteins from blood into CSF of sheep fetuses (57-86 days gestation) was studies.	CSF:plasma ratios were 15% for hAFP, 10% for hTransferrin and *sheep* albumin, 7% for hα_1_-antitrypsin, and 5% for hAlbumin. Reduced penetration of protein from blood into CSF in older fetuses. The immature choroid plexus may allow for transcellulare protein movement, important for some aspects of brain development.
Dziegielewska et al. (10)	Proteins in cerebrospinal fluid and plasma of postnatal Monodelphis domestica (gray short-tailed oposum).	Comparative Biochemistry and Physiology Part B: Comparative Biochemistry. 1989	CSF and plasma protein concentration measured from birth to adulthood.	Total protein in CSF increased from birth to a peak concentration between 5-10 days post-partum. CSF-brain barrier appears to exclude CSF protein from brain extracellular space.
Dziegielewska et al. (10)	Proteins in cerebrospinal fluid and plasma of the pouch young tammar wallaby (Macropus eugenii) during development.	Comparative Biochemistry and Physiology Part B: Comparative Biochemistry. 1986	CSF and plasma protein concentration measured from birth until leaving the pouch.	Total protein in CSF increased from birth to a peak concentration between 15-20 days post-partum.
Mack et al. (11)	Relationship between orthogonal arrays of particles and tight junctions as demonstrated in cells of the ventricular wall of the rat brain	Cell and tissue research. 1987	Ependymal cells in the ventricular wall and circumventricular organs compared with freeze-fracturing.	Ependymal cells have orthogonal arrays of particles (OAP), but not tight junctions. Choroid plexus cells present tight junctions, but not OAPs. In the boundary zone between choroid plexus and ependyma both OAPs and tight junctions coexist.
Gotow and Hashimoto (12)	Intercellular junctions between specialized ependymal cells in the subcommissural organ of the rat.	Journal of Neurocytology. 1982	Permeability of ependymal cells studied with freeze-fracturing and tracer experiments with horseradish peroxidase (HRP).	One or two strands with interruptions in the apical portion of ependymocytes. Intraventricularly infused HRP leaks through junctions but is sometimes stopped. Intercellular spaces of different lengths are found between tight junctions.
Rieke et al. (13)	Ultrastructure of ependymal cells in primary cultures of cerebral cortex.	Journal of neuroscience research. 1987	Electron microscopy of primary ependymal cultures.	Zonula occludens and adherens, membrane interdigitations were noticed in the lateral cell membrane.
Whish et al. (14)	The inner CSF–brain barrier: developmentally controlled access to the brain *via* intercellular junctions.	Frontiers in neuroscience. 2015	Study of permeability of CSF-brain barrier from embryonic day 17 until adult with multiple probes and proteomic analysis.	At early fetal stages solute movement is restricted to the smallest molecules 286Da, while by postnatal day 20 70kDa probes can diffuse freely. Gap junctions and claudin-11 were found only in adults, while N-cadherin, β - and α-catenin were detected in embryos.
Mochida et al. (15)	A homozygous mutation in the tight-junction protein JAM3 causes hemorrhagic destruction of the brain, subependymal calcification, and congenital cataracts.	The American Journal of Human Genetics. 2010	Homozygosity mapping and gene sequencing.	Mutation in splice-donor site of intron 5 of *JAM3*, chromosome 11q25. Responsible for a familial syndrome with hemorrhagic destruction of the brain, subependymal calcification, and congenital cataracts secondary to tight junction and ependymal destruction.
Drielsma et al. (16)	Two novel CCDC88C mutations confirm the role of DAPLE in autosomal recessive congenital hydrocephalus.	Journal of medical genetics. 2012	Homozygosity mapping and whole exome sequencing in two families with non-syndromic hydrocephalus	Homozygous mutation in the DAPLE encoding for CCDC88C. Truncated extreme C-terminus of DAPLE that binds Dlg1 and zo-1, with ependyma collapse.
Saugier-Veber et al. (17)	Hydrocephalus due to multiple ependymal malformations is caused by mutations in the MPDZ gene.	Acta neuropathologica communications. 2017	Post-mortem homozygosity mapping and whole exome sequencing in 3 fetuses.	Three novel homozygous null mutations in the *MPDZ* gene in fetuses with multiple ependymal malformations. MPDZ is a component of tight junctions of the ependyma/choroid plexus.
Feldner et al. (18)	Loss of Mpdz impairs ependymal cell integrity leading to perinatal-onset hydrocephalus in mice.	EMBO molecular medicine. 2017	Generation of mouse models of *Mpdz* deletion.	*Mpdz* gene deletion/conditional inactivation in Nestin-positive cells in mouse models led to hydrocephalus. Ependymocytes with normal tight junctions, but diminished expression of the planar cell polarity protein Pals1. Ependymal denudation with gliosis and aqueductal stenosis/hydrocephalus.
Yang et al. (19)	Murine MPDZ-linked hydrocephalus is caused by hyperpermeability of the choroid plexus.	EMBO molecular medicine. 2019	MRI and comparative proteomic analysis of CSF content in normal and MPDZ LOF animals.	Humans and mice with a truncated version of MPDZ develop severe hydrocephalus and death. Contrast penetration is noticed on MRI in animals with MPDZ loss of function, with increased transcytosis and paracellular permeability.
Petrov et al. (20)	Distribution of the tight junction-associated protein ZO-1 in circumventricular organs of the CNS.	Molecular brain research. 1994	Study of immunofluorescent distribution of ZO-1 in murine circumventricular organs.	Unbroken ZO-1 distribution in specialized ependymal cells adjacent to organum vasculosum laminae terminalis and subcommissural organ. Heterogeneous ZO-1 staining pattern in blood vessels and ventricular walls.
Kang et al. (21)	Effect of estrogen on the expression of occludin in ovariectomized mouse brain.	Neuroscience letters. 2006	Expression of occludin in ovariectomized female brain was studied with IHC, WB and mRNA sequencing.	Decrease in occludin expression in ovariectomized mice. 17β estradiol up-regulates occludin mRNA levels.
Steinemann et al. (22)	Claudin-1,-2 and-3 are selectively expressed in the epithelia of the choroid plexus of the mouse from early development and into adulthood while claudin-5 is restricted to endothelial cells.	Frontiers in neuroanatomy. 2006	Study of claudin expression in the choroid plexus and ependymal regions around the plexuses *via* IHC with novel fixation and alkaline phosphatase detection system.	Significant claudin-1, - 2, - 3 expression in choroidal cells and surrounding ependyma.
Alvarez and Teale (23)	Differential changes in junctional complex proteins suggest the ependymal lining as the main source of leukocyte infiltration into ventricles in murine neurocysticercosis.	Journal of neuroimmunology.	Analysis of junctional complexes of animals infected with *Mesocestoides corti*.	Reduction in ependymal occludin expression with increased leukocyte and microbial transmigration and infection spread.
Oliver et al. (24)	Disruption of CDH2/N-cadherin-based adherens junctions leads to apoptosis of ependymal cells and denudation of brain ventricular walls.	Journal of Neuropathology & Experimental Neurology. 2013	Blockage of N-cadherin function in cellular and mouse models.	Disruption of zonula adherens, abnormal intracellular accumulation of N-cadherin, ependymal denudation and obstructive hydrocephalus.
Hatta et al. (25)	Spatial and temporal expression pattern of N-cadherin cell adhesion molecules correlated with morphogenetic processes of chicken embryos.	Developmental biology. 1987	Immunohistochemistry for N-cadherin in chicken embryos.	Disappearance of N-cadherin is correlated with rearrangement, segregation, or association of cells. N-cadherin expression is related to L-CAM cellular expression.
Gänzler-Odenthal et al. (26)	Blocking N-cadherin function disrupts the epithelial structure of differentiating neural tissue in the embryonic chicken brain.	Journal of Neuroscience. 1998	N-cadherin blockage during early chicken brain development.	Invaginations of ependymal lining, formation of neuroepithelial rosettes and multiple ependymal layers in the tectum and dorsal thalamus
Sival et al. (27)	Neuroependymal denudation is in progress in full-term human fetal spina bifida aperta.	Brain pathology. 2011	Immunostaining for ependymal markers (caveolin 1, βIV-tubulin, S100), junction proteins (N-cadherin, connexin-43, NCAM), blood vessels (Glut-1) and astrocytes (GFAP) in control and Spina Bifida Aperta fetuses.	Four stages of ependymal denudation and failure were observed in Spina Bifida patients, with formation of pseudorosettes. Abnormalities in gap and adherent junctions cause defective ependymal coupling, desynchronized ciliary beating and ependymal denudation.
Guerra et al. (28)	Defects in cell-cell junctions lead to neuroepithelial/ependymal denudation in the telencephalon of human hydrocephalic fetuses.	Cerebrospinal Fluid Research. 2010	Immunocytochemistry with antibodies against N-cadherin and connexin-43, bIV-tubulin bIII-tubulin markers in hydrocephalic and SBA fetuses.	Denuded and altered ependymal areas associated with abnormal N-cadherin expression, rosettes and periventricular heterotopias.
Nechiporuk et al. (29)	Failure of epithelial tube maintenance causes hydrocephalus and renal cysts in Dlg5–/– mice.	Developmental cell. 2007	RT-PCR and sequencing of Napa in *hyh*-mutated mice.	*hyh* mice carry a mutation in Napa encoding soluble NSF attachment for αSnap. This leads to abnormal localization of E-cadherin, β-catenin, atypical protein kinase C (aPKC) and INADL.
Jiménez et al. (30)	A programmed ependymal denudation precedes congenital hydrocephalus in the hyh mutant mouse.	Journal of Neuropathology & Experimental Neurology. 2001	Immunocytochemistry and scanning electron microscopy of *hyh* mice at different stages of development.	Ependymal denudation and aqueductal obstruction starting at embryonic day 12. The authors hypothesized the onset of abnormalities in cell adhesion molecules.
Páez et al. (31)	Patterned neuropathologic events occurring in hyh congenital hydrocephalic mutant mice.	Journal of Neuropathology & Experimental Neurology. 2007	Hyh mice studied with lectin binding, bromodeoxyuridine labeling, immunochemistry, and scanning electron microscopy.	E12 ependymal denudation triggered proliferation of neighboring astrocytes. Abnormalities of the corpus callosum and hippocampal commissure. Alterations developed when hydrocephalus was not yet patent, suggesting *hyh* causes alterations in neural development.
Roales-Buján et al. (32)	Astrocytes acquire morphological and functional characteristics of ependymal cells following disruption of ependyma in hydrocephalus.	Acta neuropathologica. 2012	Electron microscopy, immunocytochemistry (N-cadherin, connexin 43, aquaporin 4, caveolin-1, EEA1. HRP and lanthanum nitrate used to track transcellular and paracellular routes.	Astrocytes share several features with normal ependyma, such as microvilli, gap junctions, expression of aquaporin 4, caveolin-1 and EEA-1. The also show similar behavior in the paracellular route of molecules/water between CSF, the subependymal neuropile and pericapillary space.
Baeza et al. (33)	IIIG9 inhibition in adult ependymal cells changes adherens junctions structure and induces cellular detachment.	Scientific reports. 2021	Expression and localization of IIIG9 in the adherens junctions (cadherin/β-catenin-positive junctions) of ependymal cells with confocal and electron microscopy.	Ependymal cells with a “balloon-like” morphology. Reduced cadherin, cleavage of caspase-3, “cilia rigidity” and ventriculomegaly occurring prior to these events. IIG9 is essential for the maintenance of adherens junctions.
Klezovitch et al. (34)	Loss of cell polarity causes severe brain dysplasia in Lgl1 knockout mice.	Genes & development. 2004	Histology and immunohistochemistry of Lgl1 knockout mice.	Loss of Lgl1 in mice with formation of neuroepithelial rosettes. Lgl1–/– neural progenitor cells fail to exit the cell cycle and die by apoptosis. Mice develop hydrocephalus and die. This may be explained by incorrect localization of Numb, inhibitor of Notch.
Imai et al. (35)	Inactivation of aPKCλ results in the loss of adherens junctions in neuroepithelial cells without affecting neurogenesis in mouse neocortex.	Development. 2006	Nestin-Cre mediated conditional gene targeting system in aPKCλ ko mice.	Loss of adherens junctions, retraction of apical processes and impaired interkinetic nuclear migration in ependymal cells at E15. Neurogenesis was not affected.
Ma et al. (36)	Loss of cell adhesion causes hydrocephalus in nonmuscle myosin II-B–ablated and mutated mice.	Molecular biology of the cell. 2007	Ablation of NM II-B or replacement with decreased amounts of mutant (R709C), motor-impaired form in mice	Mesh-like structure present at the apical border of ependymocytes containing NM II-B, β-catenin and N-cadherin, with a role in cell adhesion. Mesh-like structure, canal patency and hydrocephalus can be restored by increasing expression of NM II-B.
Tullio et al. (37)	Structural abnormalities develop in the brain after ablation of the gene encoding nonmuscle myosin II-B heavy chain.	Journal of Comparative Neurology. 2001	Ablation of nonmuscle myosin heavy chain II-B (NMHC-B) in mice.	Severe congenital hydrocephalus secondary to disruption of the ventricular surface and cell migration. Formation of rosettes and aqueductal obstruction.
Bátiz et al. (38)	Heterogeneous expression of hydrocephalic phenotype in the hyh mice carrying a point mutation in α-SNAP.	Neurobiology of disease. 2006	*hyh* mouse carrying a mutation for α-SNAP and light and electron microscopy.	70% of animals with rapidly progressing hydrocephalus, 30% with slowly progressing hydrocephalus and spontaneous ventriculostomies between the ventricles and the SAH that allowed them to survive.
Bátiz et al. (38)	A simple PCR-based genotyping method for M105I mutation of alpha-SNAP enhances the study of early pathological changes in hyh phenotype.	Molecular and cellular probes. 2009	High-throughput genotyping of *hyh* mice, to correlate genotype-phenotype, the earliest pathological changes of hyh mutant mice.	Single-gene autosomal recessive disorder with 100% penetrance causing altered membrane trafficking and hydrocephalus.
Fabbiani et al. (39)	Connexin signaling is involved in the reactivation of a latent stem cell niche after spinal cord injury.	Journal of Neuroscience. 2020	Patch-clamping of ependymal cells with ICH for connexins in neonatal and adult normal vs. injured spinal cord mice.	Coupling decreases with postnatal development but increases again after SCI. increase in connexin 26, proliferation and reduction of connexin hemichannel activity were also noticed in the spinal central canal.
Bouillé et al. (40)	Gap junctional intercellular communication between cultured ependymal cells, revealed by lucifer yellow CH transfer and freeze-fracture.	Glia. 1991	Study of gap junctions in primary cultures from fetal mouse or rat hypothalamus and choroid plexus. Transfer of Lucifer Yellow CH after intracellular microinjection and freeze-fracture.	Gap junctional transfer of dye present in ciliated ependymal cells, choroidal ependymocytes and non-choroidal ependymocytes. No dye movement in astrocytes.

First author's name, article's title, journal, methodology used, and main findings are presented in the table. Papers are subdivided by type of junction investigated. Multiple papers focused on more than one junction type—assignment was based on the main type of junction found by the investigators. Gray, non-specific cellular junctions; yellow, tight junctions; blue, adherens junctions; green, gap junctions.

### 2.1. Ependymal structure and permeability: location- and age-related changes in structure and permeability

Extensive evidence has been gathered over the last decades on the composition and role of the blood-brain and blood-CSF barriers, at the capillary-astrocytic level and in the choroid plexuses (42–46). Much less is known for the CSF-brain interface at the ependymal level, despite its significant surface area and close relationship to the parenchyma and deep nuclei of the brain. The ependymal layer, despite the general belief, provides a regulated barrier to the diffusion of solutes and water between the CSF and interstitial compartments. Both regionality and age play a significant role in determining the magnitude and selectivity of this route.

#### 2.1.1. Regional variability

The first data on the intercellular junctions at this location was gathered using electron microscopy, starting in the 1960s, when Tennyson and Pappas reported the presence of highly elaborated, thin cytoplasmic plates or finger-like projections emanating from the ependymal cells covering the aqueduct of rabbit models (4). Duckett et al. noticed that the arrangement of the germinal layer cells resembled that of other absorptive units such as the renal proximal tubules, the gallbladder and the intestine, and suggested an absorptive function for the ependyma (5, 47). Rieke et al. investigated the ependymal ultrastructure of embryonic rat cells (gestational day 14–17) *via* electron microscopy. Again, their observations confirmed the expression of zonula occludens and adherens in the apical third of the cell membrane, as well as membrane interdigitations (13).

A significant variability between different areas of the ventricular lining is present. While choroid plexus epithelia have fully formed apical tight junctions that constitute the blood-CSF barrier (46), circumventricular organs show hybrid characteristics. The choroid plexus is characterized by welldeveloped pentalaminar tight junctions, whereas the ependyma presents only “labile” and non-overlapping rows of morphologically distinguishable appositions that do not interfere with the intercellular movement of tracers (8). Local expression of apical claudin-3 as well as orthogonal arrays of particles have been described along the circumventricular organs, with a notable reduction in horseradish peroxidase diffusion compared to the surrounding regions (11). Similar findings were reported by Altafulla et al. in a rat model of intraventricular sucrose injections. While significant and rapid movement from the lateral ventricles across the ependymal layer was noticed, a slower rate of diffusion was present in the circumventricular organs, medullary and cerebellar tissue, superior and inferior colliculi and cerebral peduncles (48).

Other examples of self-regulating capacity come from local injection of reagents and carbohydrates. Ependymal, but not choroidal, junctional damage with phorbol ester is able to reduce the movement of biotinylated dextran by modulating protein kinase C activity (49). Kuchler et al. also showed that intraventricularly-injected mannose-containing neoglycoproteins induced the disappearance of intercellular junctions (50).

#### 2.1.2. Age-dependent variability

Several works have focused on age-dependent evolutions in tight and “strap” junctions (51). Cavanagh and Warren investigated the movement of endogenous vs. sheep albumin using a rodent model of CSF flow. Both proteins were detected in the brain parenchyma starting on day 16–17 of gestation, but not earlier, mimicking the changes in tight junction appearance seen in other studies with contrast tracers. Further, transcellular movement of albumin into the ependymal cells and sub-ependymal layer of the cortex was described, suggesting the existence of a specialized mechanism of uptake rather than paracellular unrestricted diffusion (9).

Fossan et al. determined that extracellular penetration of several millimeters from the ependyma is present in older sheep fetuses and adults. Partial limitation of brain parenchyma diffusion was described in younger fetuses, with a steady increase in barrier permeability with age of both radioactive ([^3^H]inulin, [^14^C]sucrose, [^125^I]albumin) and fluorescent (horseradish peroxidase) markers (6). Møllgård et al. studied gap and tight junctions in a sheep model of the ventricular zone germinal matrix. With freeze-fracture electron microscopy they were able to describe not only spirals of zonulae adherens junctions, but also large gap junctions extending from the ventricular pole along the lateral cell membrane (7).

Mori and Kazanis demonstrated that neuroepithelial cells of the neural tube are connected by gap, adherens and tight junctions until embryonic day 12 (E12), but that tight junctions regress and disappear from E12 onward (52, 53). A genetic or cellular insult can therefore affect neural and ependymal lining integrity if delivered at this point in the neural tube development (54).

Age-related changes in ependymal permeability are also suggested by the pathophysiology of several hydrocephalus models. These confirmed that junctional proteins are involved in ependymal stability, and that alterations in brain and ventricular development result from their mutation. The *hyh* and other models characterized by cell-cell junctional defects have shown abnormal flow of CSF, VZ disruption and eventually development of hydrocephalus (37, 38, 55, 56) (Table 2).

**Table 2:** Main proteins identified in the ependymal intercellular junctions.

	**Tight junctions**	**Adherens junctions**	**Gap junctions**	**Associated proteins**
**Ependymal junctional proteins by age**				
Embryonic Ependyma 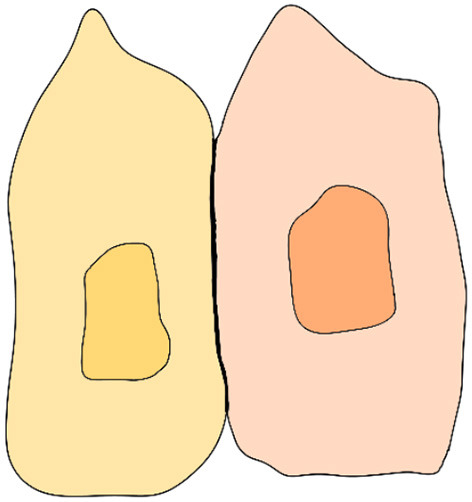	**Claudin-5** (E17) (14)	**N-cadherin** (E17) (14) **Protocadherin** **γ-C3** (E17) (14) **T-cadherin** (E17) (14)		**β-catenin 1** (AJ) (E17) (14) **β-catenin 2** (AJ) (E17) (14) **α-catenin 1** (AJ) (E17) (14) **β-catenin-interacting protein 1** (AJ) (E17) (14) **δ-catenin-2** (AJ) (E17) (14)
Adult Ependyma 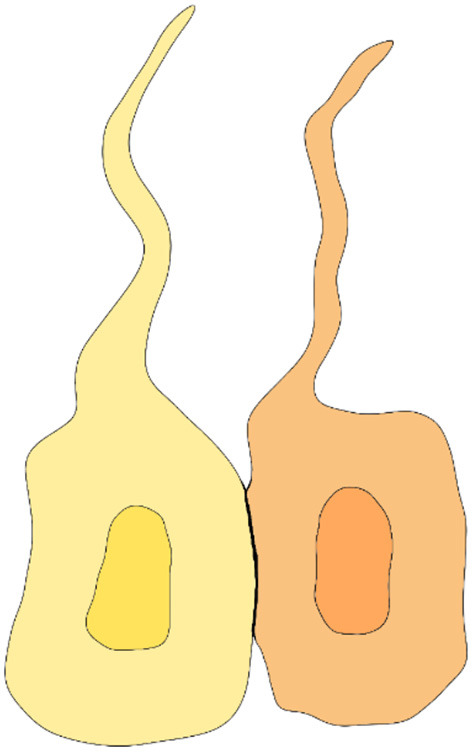	**Claudin-11** (14) **Claudin-1** (22) **Claudin-2** (22) **Claudin-3** (22) **Occludin** (52) **Jam-2** (14) **Jam-3** Subependymal calcifications, hemorrhagic destruction of the brain, hydrocephalus (15). **ZO-1** (20)	**Plakophilin-4** (14) **N-cadherin** Hydrocephalus, ependymal denudation, SBA, deficiency of Cx43 and intracellular trafficking (24–28). **E-cadherin** **Protocadherin** **γ-C3** (14)	**Connexin-29** (14) **Connexin-30** (57) **Connexin-32** (14) **Connexin-43** (58) **Connexin-26** (spinal ependyma) (112)	**MDPZ** (TJ) Congenital hydrocephalus with ependymal hydrocephalus (17–19). **β-catenin 1** (AJ) (14) **α-catenin 1** (AJ) (14) **δ-catenin-2** (AJ) (14) **α-catenin 2** (AJ) (14) **Dlg5** (AJ) Failure of t-SNARE dependent vesicular trafficking, loss of catenin-cadherin adhesion and cellular orientation (29). **αSNAP** (AJ) Failure of vesicular trafficking and loss of E-cadherin (38). **IIIG9** (AJ) Deletion causes hydrocephalus and ependymal denudation (33). **Nonmuscle Myosin II-B** (AJ) Hydrocephalus and ependymal denudation (36).

Proteins are divided by developmental age (embryonal vs. adult) and function. The main clinical correlates are listed for each junctional element. A schematic of the ependymal changes during embryonic and adult life is provided in the left column. AJ, adherens junction; TJ, tight junction.

**Figure 2:**
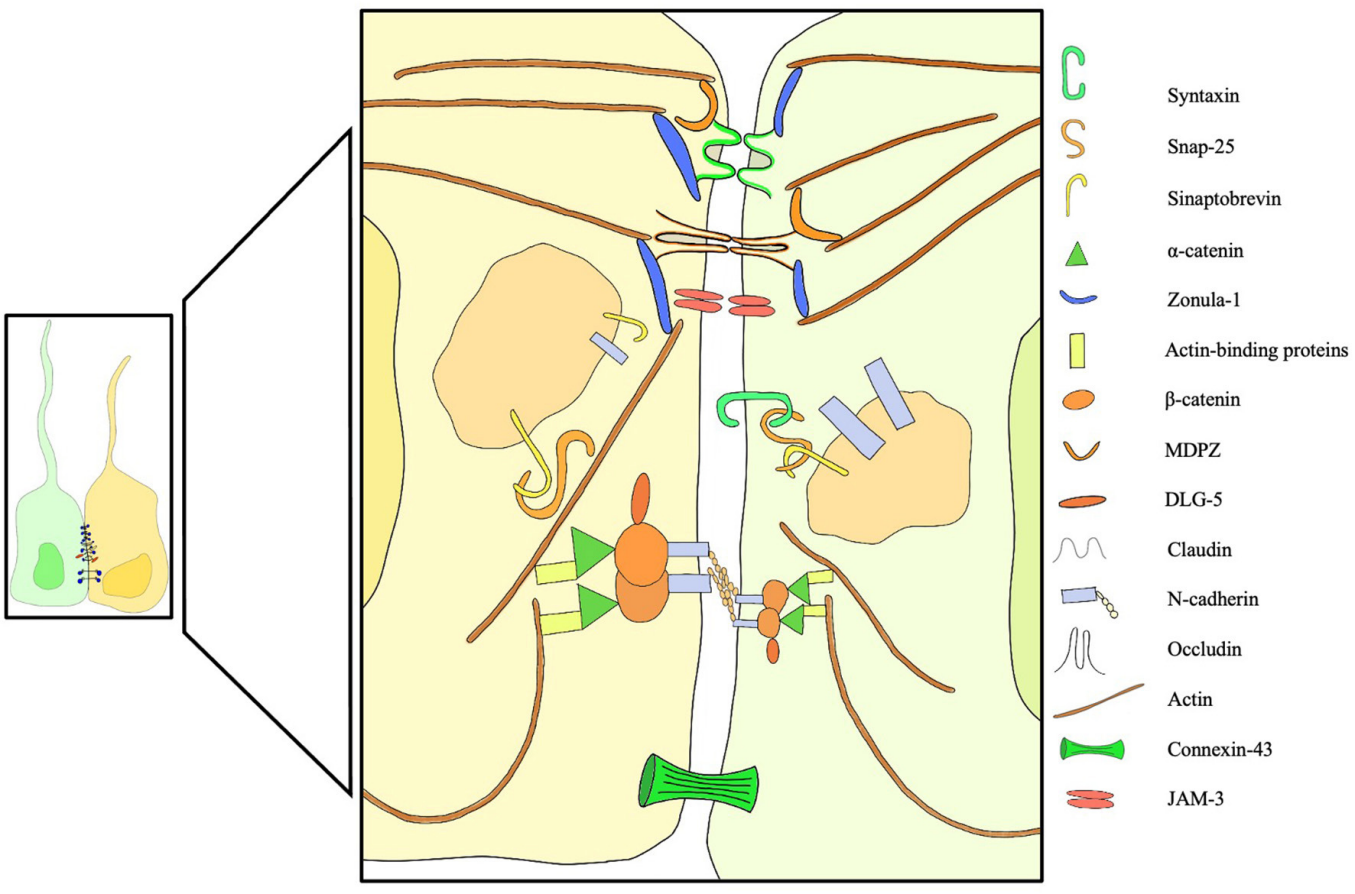
Overview of membrane, trafficking, junctional, cytoplasmic structural proteins involved in intercellular junctions and pathological processes of the ependyma. Failure of one of these components can induce ependymal denudation and hydrocephalus in murine models. A dedicated legend is provided with the figure.

In recent years, with the advent of fluorescent microscopy, novel dextran-based tracers and mRNA sequencing, the expression profile of intercellular junctions has finally started to become clearer. Whish et al. (14) provided the first evidence of age-related changes in protein and gene expression during the transition from the embryonic ventricular zone to the adult ependyma. Interestingly, a significant limitation to the passage of larger tracers was noticed early in ependymal development, possibly part of a defensive mechanism that protects the developing brain tissue from high CSF protein concentrations (14). Some suggest that the barrier function plays an important role in restricting access to the subependymal brain tissue during fetal life (10, 59–65), protecting the brain from high protein concentrations that would cause developmental impairment and tissue damage (66). Others hypothesize that the high colloidal pressure functions as a tool to drive water into the ventricles (66–68), creating a hydrostatic pressure gradient necessary for cortical gyral growth (Figure 2).

### 2.2. Tight junctions

Tight junctions are a central component of non-permeable epithelia, allowing cells to create a physical barrier between two compartments. Also called zonula occludens, these junctions are found in epithelial and endothelial cells and regulate the passage of cells, solutes and water. Usually located in the apical portion of the cell membrane, tight junctions structurally occlude the intercellular space between adjacent cells, creating an impermeable belt that spans the entire epithelium (69). Single- and four-pass transmembrane proteins, such as claudins, occludins and JAMs (junctional adhesion molecule), constitute the bulk of this class of proteins (69). Despite the evidence of belt-like apical junctional complexes in ependymal cells that was reported by several groups starting in the 1960s (4, 7), the significant permeability of this cellular layer to inulin, sucrose, albumin and horseradish peroxidase suggested a limited and morphologically incomplete expression of these junctions at the ependymal level (6). Recent studies shed light on several components of this class of proteins, suggesting a significant degree of plasticity during the first weeks of life and a prominent role in shaping membrane permeability to solutes and water.

#### 2.2.1. Claudins

The presence of a developmentally-regulated CSF–brain barrier was first hypothesized in the 1960s, when “strap junctions” were identified between neuroepithelial cells in early stages of development *via* electron microscopy. This function may play an important role in restricting access of proteins, present in high concentration in fetal CSF, to the brain parenchyma during neurodevelopment. A study examining the cellular distribution of native and foreign albumins injected into fetal and neonatal ventricles of rats further supported this hypothesis (9).

Recently, this interface has been the object of several studies with lipid insoluble markers that used a wide range of molecular weights, in conjunction with the rebirth of CSF circulation studies and discovery of the glymphatic system. In a recent paper, Whish et al. found that the barrier is permeable only to smaller molecules during early embryonic life and that the diffusional restraint is removed progressively during fetal and post-natal life (14). Specifically, the diffusion of molecules >3 kDa was limited at E17 and E19 but resumed at P0. Similar results were noticed for tracers with a weight >10 kDa. No significant difference in CSF protein concentrations was found between fetal and P0 animals, while a lower concentration was measured starting from P2. RNA sequencing was also performed during fetal and adult age, showing a switch from Claudin-5 expression to Claudin-11 between fetal and adult animals. These molecular changes may explain the higher permeability of the ependymal layer in adult animal models, providing important information for future studies in intercellular permeability. Most importantly, this evidence confirmed the existence of a molecular change at the ependymal level.

Other structures formed by multiple intramembrane particles such as aquaporin-4 and other junctional proteins are the orthogonal arrays of particles (OAP). OAPs are usually found in the glia limitans, but have also been described in the ependymal layer and in striated muscle, kidney, and stomach (70, 71). Mack et al. suggested the existence of a transitional zone between the ventricular ependyma and choroid plexus where the density of OAP is low and tight junctions are well developed. OAPs are usually prevalent in the ependymal layer, while tight junctions predominate in the choroid plexus (11). Gotow and Hashimoto also found one or two relatively continuous strands of tight junctions with interruptions in the apical portion of ependymocytes in the rat subcommissural organ. HRP infusions left parts of the intercellular spaces between membrane fusions of tight junctions unstained. Nonetheless, the intercellular space below the junction is usually stained by the enzyme, testifying dye movement across the ependymal lining (12).

Other members of the claudin superfamily such as claudin-1, - 2, - 3 were described in detail in the lateral and fourth ventricle choroid plexus, playing a role in fluid and ion secretion. Interestingly, these proteins appear to also line the choroid and ependymal epithelia, as well as the circumventricular organs, probably contributing to trans-ependymal flow of CSF and ions (22).

#### 2.2.2. Occludin

Occludin, the first transmembrane protein localized at the TJ, shares the tetraspan structure with cytoplasmic N- and C-termini with claudins. Initially thought to contribute barrier function, as well as cellular orientation, paracellular flow and lipid segregation (72), its role has been expanded in recent years to intracellular signaling *via* the PI3K pathway (73). Further, it is expressed in the brain vascular epithelium and has recently been connected with a congenital form of simplified gyration and polymicrogyria (15, 74).

Interestingly, upon development of parasitic ventricular infection, ependymal occludin expression appears to be reduced, allowing for increased leukocyte and microbial transmigration, as well as infection spread (23). A recent study also highlighted the changes in TJ expression secondary to variations in estrogen levels in female rodents. Kang et al. suggested the presence of occludin in murine ependyma and showed a significant decrease in expression in ovariectomized mice. Further, 17β estradiol was noticed to up-regulate occludin mRNA levels in these animals (21).

#### 2.2.3. Jam-3

Junctional adhesion molecules (JAMs) are part of the immunoglobulin superfamily involved in a number of cellular processes, from tight junction assembly, to angiogenesis, leukocyte transmigration, platelet activation (75). Their function might be related to structural or barrier integrity, and recent evidence points toward higher expression of Jam-2 and Jam-3 in adult compared to embryonic rodents (14). A novel mutation in the Jam-3 gene was recently identified in a consanguineous family (15). Subependymal calcifications and hydrocephalus, as well as hemorrhagic destruction of the brain parenchyma were reported, suggesting a possible role in ependymal stability (15).

#### 2.2.4. MDPZ

MPDZ is a scaffold protein of the apical complex responsible for junctional stability and frequently bound to other scaffolding proteins such as DAPLE (16, 76). Interestingly, these proteins are involved in non-syndromic forms of congenital hydrocephalus. Specifically, disruption of TJs secondary to abnormal cell-cell adhesion by MPDZ (also named MUPP1) has recently been reported in several fetuses with congenital hydrocephalus. The mechanism by which MDPZ alters CSF dynamics is likely secondary to uncontrolled production of fluid and ependymal denudation with obstruction of the aqueduct and subarachnoid spaces (17).

Feldner et al. (18) further investigated the role of MDPZ in CSF ependymal dynamics by generating mouse models with global MDPZ gene deletion. These animals showed inactivation of Nestin-positive cells, with development of hydrocephalus in the postnatal period. Despite showing morphologically normal ependymal cells, MDPZ-deficient animals had lower expression of the interacting planar cell polarity protein Pals1 and progressive loss of barrier integrity. Further, ependymal denudation was observed, together with reactive astrogliosis and aqueductal stenosis, suggesting a role for tight junctions in ependymal structural integrity (18). MRI studies have also confirmed contrast percolation in mice carrying a MDPZ loss-of-function mutation. CSF proteomic analysis of normal and MDPZ null hydrocephalic mice showed a 53-fold increase in protein concentration. This evidence suggested higher choroidal and ependymal transcytosis of fluid and solutes, explaining the development of hydrocephalus following MDPZ loss-of-function (19).

#### 2.2.5. Zonula Occludens-1

Zonula Occludens-1 (ZO-1) is part of a family of three structurally related proteins, ZO-1, ZO-2 and ZO-3 that mediate protein-protein interactions downstream of tight and adherens junction complexes. Specifically, ZO-1 is responsible for binding claudins and JAMs, as well as the adherens junction proteins afadin and α-catenin, connecting these structures to the actin cytoskeleton (77–83). Petrov et al. (20) investigated the role of this protein in murine circumventricular organs using immunofluorescence techniques, showing a punctate pattern of ZO-1 distribution, suggestive of discontinuous tight junctions at the circumventricular and ependymal level. Interestingly, continuous ZO-1 distribution was observed in the areas adjacent to the organum vasculosum of the laminae terminalis and the subcommissural organ, probably a sign of unbroken tight and/or adherens junctions in these regions (20).

### 2.3. Adherens junctions

#### 2.3.1. Cadherins

Cadherins are the major Ca^2+^-dependent families of junctional proteins present in the brain, and a central component of the ependymal tight junctions. First described in the 1980s in ultrastructural studies of the ependymal cell, their position and role have been the object of several studies in animal models and human tissue. Interestingly, both N- and E- cadherins co-exist at the ependymal level, mediating similar functions at the ependymal cell border and playing a fundamental role for structural integrity. Further, they appeared to be responsible for ependymal denudation and hydrocephalus when mutated. Several junction-associated proteins and downstream mediators, such as β-catenins, p120 and paxillins were also stained on the cytoplasmic surface. Phosphotyrosine immunoreactivity points toward the presence of several other proteins involved in downstream signaling, suggesting a role for adherens junctions in determining cell orientation (84). Rieke et al. described adherens junctions as a distinct intercellular space between membranes with dense thickening of the cytoplasmic surface, spanning a significant area on apposed cells (13). Importantly, cadherins appear to play a central role for ependymal structural stability and cell integrity, as suggested by several studies in congenital hydrocephalus models.

#### 2.3.2. N-cadherin

Oliver et al. (24) showed that blocking N-cadherin function with specific peptides that target the histidine-alanine-valine extracellular homophilic interaction domain can cause disruption of the zonula adherens and abnormal intracellular accumulation and sequestration of N-cadherin. This precedes ependymal apoptosis and disruption, with final generalized denudation. Significant reduction in the extension of adherens junctions and development of asymmetrical distribution was noticed after inhibition with HAV-peptide. Interestingly, ependymal cells start undergoing apoptosis before denudation, with ~80% of cells positive on TUNEL test after 24 h from treatment with HAV-peptide. These changes take place in absence of hydrocephalus, increased ICPs or stretch forces, and suggest that cadherins and adherens junctions not only have a structural function, but also play a role in cell survival and apoptosis when the intercellular contacts weaken (24).

The inhibition of N-cadherin in chick embryos induces the formation of ependymal rosettes and periventricular abnormalities (25, 26, 84). Human studies in control and spine bifida aperta (SBA) embryos found that loss of N-cadherin leads to ependymal denudation, formation of pseudorosettes and macrophage invasion. Further, a concomitant loss of the gap junction protein connexin 43 was noted in some of these samples (27), and while other junctional proteins were noted, small rosettes did not express N-cadherin (27). Sival et al. (27) and Guerra et al. (28) hypothesized that the abnormal subcellular localization of N-cadherin and connexin 43 may be explained by altered intracellular trafficking, with intracytoplasmic buildup of both proteins. This would result in a defect in cell-to-cell adhesion, ependymal denudation and aqueductal obstruction with ventriculomegaly (27, 28).

#### 2.3.3. E-cadherin

E-cadherin is another central component of Ca^2+^-mediated cell-to-cell adhesion, and plays a role in ependymal development and integrity by regulating neural stem cell proliferation (85). Several intracellular pathways are involved in mediating E-cadherin expression. Among others, Numb-mediated signaling appears to be involved in cell border E-cadherin localization during ependymal maturation, and its disruption leads to epithelial damage *via* Notch-mediated signaling. VEGF seems to also play a role in ventriculomegaly, as shown by several studies (86–88). Interestingly, part of this effect might be due to the reduction in E-cadherin expression and ependymal denudation induced by VEGF administration. These findings are reversed with anti-VEGF antibody use (89).

Several studies have suggested a role for intracellular vesicular trafficking in determining junctional integrity. Failure of cadherin delivery to the cell surface could therefore explain ependymal denudation and hydrocephalus in rodent models.

Dlg5, a protein involved in t-SNARE-dependent movement of vesicles to the cell membrane, is thought to affect delivery of cadherin-catenin adhesion complexes and cellular orientation. Further, this protein appears to play a central role in epithelial tube maintenance in kidney and brain development. With loss of cell polarity, hydrocephalus and renal cysts may develop, as shown in Gld5-/- mice by Nechiporuk et al. (29).

αSNAP is another protein involved in membrane fusion events, thought to be responsible for the *hydrocephalus with hop gait* (*hyh*) phenotype. Mice homozygous for this deletion develop hydrocephalus with 100% penetrance, as well as ependymal damage and denudation (56). Point mutations in αSNAP appear to be sufficient to induce ventricular denudation and hydrocephalus in this model (38). Further, animals carrying mutations in SNAP-receptor (SNARE) mediated vesicle fusion system exhibit defects in apical protein localization, as well as errors in cell fate determination in neuroepithelial cells, with consequent cortical neuronal depletion (90). Chae et al. (90) described the role of αSnap mutation in *hyh* mice. This trafficking intracellular protein is suspected to be responsible for the movement of N-cadherin to the cell membrane. An alteration in this process leads to N-cadherin failure with collapse of the membrane and ependymal integrity.

Jimenez et al. (30) have shown that ependymal denudation precedes and triggers the development of obstructive hydrocephalus in a *hyh* mutant mouse model. Two phases are noted in these animals. First, ependymal cells detach from the ventricular wall, and a communicating form of hydrocephalus is seen. In a second phase, aqueductal obstruction ensues, usually post-partum and after the ependymal lining has been cleared. This led the authors to hypothesize a role for a bulk flow mechanisms at the brain interstitium-CSF interface, potentiated by ependymal denudation secondary to failure of adherens junctions (30). Paez et al. (31) demonstrated that different degrees of ependymal denudation are present in the brain, with the cingular and frontal cortices being the most affected regions. Noticeably, these alterations developed at stages when hydrocephalus was not yet evident, indicating that abnormal cortical development and hydrocephalus are concomitant and explained by a similar mechanism (31). Evidence suggests that astrocytes can replenish the denuded areas, taking on some ependymal traits such as aquaporin and connexin 43 expression, re-establishing a number of lost functions at the brain-CSF interphase (32).

IIIG9, a protein restricted to ependymal cells located in the apical cytoplasm and cilia and associated to cadherin/β-catenin-positive junctions, was also studied and characterized in relation to hydrocephalus in rodents. Baeza et al. showed ependymal denudation and balloon-like morphology of ependymal cells, as well as loss of cell polarity, after IIIG9 (PPP1R32) deletion (33).

Other proteins associated with adherens junction formation and thought to have a role in VZ disruption, hydrocephalus and brain dysplasia are Lgl1 (34), atypical protein kinase C-lambda (aPKCλ) (35) and non-muscle myosin II-B (NMII-B) (36).

### 2.4. Gap junctions

Gap junctions are considered as channels that structurally interconnect ependymal elements, as well as waterways to exchange signaling molecules between neighboring cells (91–93). Connexin 43 (Cx43) in particular is thought to interact with several intracellular cascades including kinases, phosphatases, membrane receptors, cell signaling and scaffolding proteins (91). Recent evidence has shown that gap and tight junction protein expression is regulated by connexins, and co-immunoprecipitation is frequently observed (94–98). Connexins can form hemichannels that appear to be involved in cellular regulation, as well as Ca^2+^ mediated toxicity, cell proliferation and response to injury. Recent evidence has shown their involvement in several neurodegenerative pathologies such as Parkinson's disease, glioma, cerebral ischemia, spinal cord injury and chronic pain (99).

A close relationship has been found between gap and adherens junction formation in epithelia throughout the body (100–106). N-cadherin and Cx43 in particular appear to co-localize to the cell membrane, and abnormalities in one usually reflect in alterations in the other's behavior and functionality (104, 107).

While initial evidence suggested that the role of gap junctions was primarily that of facilitating ciliary movement, synchronizing their beating and therefore CSF movement, recent evidence hypothesized a broader role for these connecting junctions. Cx43 helps integrating the ependymal layer with the underlying glia, possibly regulating water and ion transport across these layers and into the interstitium and circulation (58). The abundance of gap junctions in the ventricular ependyma, glia limitans and surrounding neurons suggests the existence of a functional panglial syncytium that regulates brain-CSF interactions across broad regions of the neuraxis (58).

Gap junctions may play a major role in synchronizing the beating of ependymal cilia (108–110) and directing CSF flow in the lateral ventricle and down the cerebral aqueduct (111–113) in several mammalian and non-mammalian models of CSF circulation. Further, dysfunctional cilia have been associated with communicating ventriculomegaly, despite a thorough understanding of the underlying pathophysiological mechanism is still lacking (112).

Bouille et al. first described the existence of gap junctions in the ependymal layer in long term primary cultures derived from fetal mouse or rat (40). Different cell types were injected with Lucifer Yellow CH and analyzed with freeze-fracture. The capacity to transfer dye was very different based on cell types. Direct communication *via* gap junctions was observed among hypothalamic ependymocytes, plexal and non-plexal ependymocytes from the diencephalic roof. On the other hand, in astrocytes found in the same primary cultures, no GJIC was observed in spite of the expression of welldifferentiated gap junctions (40). This suggests a prominent functional role for gap junctions in the ependymal layer, a role that is frequently lost in hydrocephalic states (32).

Roales-Bujan et al. (32) demonstrated that wild-type and *hyh*-mutant mouse ependymal cells express connexin 43. This protein is usually located in supranuclear granules and slender patches in the lateral plasma membrane, connecting nearby cells. Further, apical localization of connexins was confirmed in this study, showing a belt-like distribution in the top third of normal ependyma, and significant disruption of this layout in *hyh* animals. As mentioned in a previous section, this was coupled with an overall decrease in N-cadherin expression and failure of intracellular transport machinery. HRP percolation into the surrounding brain parenchyma was comparable between the two groups, but its overall magnitude appeared to be somewhat controlled and regulated by the ependymal layer and the glial substitutes. Electron microscopy studies of the ependymal interdigitations showed the presence of adherens and gap junctions, while tight junctions were missing. Intraventricular injections of lanthanum penetrated through the extracellular space, filling the intercellular space of the neuropile and reached the intercellular space up to the tight junctions joining endothelial cells. Finally, after ependymal denudation, it was noticed that the reactive astrocytic layer contained Cx43 granules throughout the cytoplasm, especially in older astrocytic layers (P30 mice compared to P6) (32).

Connexin-26 was recently found to be associated with resumption of ependymal proliferation in the spinal central canal after spinal cord injury and reduction of connexin hemichannel activity. These findings were reversed by connexin inhibition (39).

## 3. Discussion

Growing evidence suggests a prominent role for disruption of the ventricular (VZ) and subventricular (SVZ) zones during embryonic life in hydrocephalus development. Cells in the VZ and SVZ give origin to most cells of the mammalian brain, including ependymal cells. These cells are part of the pseudostratified neuroepithelium that constitutes the VZ, a transient embryonic layer that contains multipotent radial glia/stem cells. Neural stem cells (NSCs) start differentiating during the fourth through the 22nd week of gestation (114), and are bathed by primordial, protein- and solute-rich CSF during early development. Recent works have focused on the junctional changes that ependymocytes undergo during the switch from early embryonic to neonatal and adult life. Until embryonic day 12 (E12) these cells are connected by gap, adherens and tight junctions (14, 52, 53). From E12 onward, tight junctions are missing or limited to the apical segments of ependymocytes, and cell-to-cell adhesion relies mainly on gap and adherens junctions. Further, a change in claudin expression, and therefore tight junction composition, was reported by several groups. These changes affect not only the movement of interstitial fluid to and from the brain parenchyma, but also the passage of solutes such as proteins and ions across these compartments.

### 3.1. Junctional proteins contribute to the paracellular flow of solutes and CSF

CSF dynamics have been studied with intraventricular, subarachnoid or lumbar injection of fluorescent and radiographic dyes for a long time. In the 1950s, studies carried out using radioactive water showed that at least part of the CSF secretion was not related to the choroid plexus (115–117). Using a rabbit model Pollay and Curl concluded that almost 30% of the total CSF secretion may come from the ependymal layer (118), while Cserr et al. showed by injecting tracers into the parenchyma that the ependymal contribution is ~1% (119).

However, in the last decade studies were able to optimize the use of fluorescent dyes for *in vivo* tracing of solute movement. Whish et al. (14) provided the first evidence of limited membrane permeability during early development, reporting an extensive change in junctional protein expression in peri- and post-natal stages and an overall increase in transependymal flow with age.

This work also suggests that ependymal permeability may be at the basis of several pathological states. Variations in CSF secretion or reabsorption can affect ventricular volume and brain function, as well as the parenchymal concentration of proteins and electrolytes. By highlighting the role of specific proteins such as Claudin-11 and−5, this group provided preliminary evidence that junctional complexes, previously thought to play a minor role in ependymal permeability, can profoundly affect CSF dynamics. Further, mRNA sequencing data was able to show that the evolution in junctional expression is paired with a direct alteration in the size of tracers able to diffuse across this barrier. This suggests that the ependyma may be a central part of the CSF secretion/absorption equation in certain phases of our lives. It also suggests that by changing the type of junctional claudin, the ependymal layer could function as a route for fluid and substance clearance.

Hypothetically, these findings suggest that paracellular junctional complexes could be modulated in order to increase or decrease the transependymal flow of water, solutes and medications. Importantly, this may constitute an additional avenue for treatment of hydrocephalus and other conditions affecting the CNS. By inhibiting certain isoforms such as claudin-5 or -11, it might be possible to impair their barrier function, prompting a more sustained centrifugal movement of water toward the interstitial compartment. This would build an additional pathway of reabsorption that is common to other mammals, but not particularly well developed in humans. On the other hand, inhibition of claudin-2, a paracellular channel for water and Na^+^ ions, could bolster fluid retention in the ventricular compartment. Finally, this lining can function as a potential route for passage of intraventricular chemo- or biological therapies. Drugs could be administered locally, and by *in vivo* modification of tight and adherens junctions clinicians could promote or limit the movement between these compartments.

### 3.2. Models of hydrocephalus secondary to ependymal denudation—Is there a common pathway from junctional failure to ependymal denudation?

Another important implication of intercellular junctional complexes lays in its role in ependymal denudation and obstructive hydrocephalus. Several rodent hydrocephalus models converge on this type of tissue damage, despite different mutations leading to the same effect. Most of these mutations involve genes responsible for the formation of gap and adherens junctions, or intracellular trafficking of their components. Among others, Chae, Batiz and Ferland focused on the intracellular movement of E-cadherin and β-catenin in *hyh*-dependent hydrocephalus (38, 54, 90), while Nechiporuk et al. suggested that Dlg5 knockout determines a deficiency in membrane assembly of cadherin-catenin complexes, leading to loss of ependymal cell polarity (29). Similarly, Rasin et al. (120) showed that Numb and NumbI inactivation disrupts intracellular transport of certain components of adherens junctions (120). Loss of adherens junction integrity was also described by Imai et al. after knockout of aPCKl in a mouse model, with derangement of ependymal cytoarchitecture (35). Klezovitch reported the onset of ventriculomegaly in animals with homozygous deletion of Lgl-1. This protein has been implicated in maintenance of cell polarity in Drosophila and mammalian cultures, and it is frequently mutated in epithelial malignancies, with loss of cell polarity and formation of pseudorosettes. Similarly, hyperproliferation and loss of polarity is seen in neuroependymal cells, with consequent development of hydrocephalus (34). Finally, Ma et al. reported ependymal collapse and aqueductal obstruction after deletion of Nonmuscle Myosin II-B in mice (36). Interestingly, increasing the local expression of this protein restored ependymal structure and function, improving CSF drainage (36).

Other studies, not directly connected to the brain ependyma, also suggest a remarkable interconnection between these proteins. ZO-1, a tight and adherens junction-associated element that links to the actin cytoskeleton, is thought to interact with Cx43 in forming gap junction plaques (121). Specifically, a failure of ZO-1 assembly results in their combined collapse. Interestingly, lack of cadherin assembly also impairs claudin and occludin formation (122).

Overall, emerging evidence suggests a central function for ependymal tight, adherens and gap junctions, and a profound interconnection that only a few years ago could not even be hypothesized. These studies show that mutations in several structural and non-structural genes result in ependymal collapse and denudation. An error in one of these proteins will likely lead to failure of the intercellular junctional system.

Another important change shared by several hydrocephalus models is the development of significant ventriculomegaly before the onset of ependymal denudation. While structural failure of the intercellular junctional proteins was thought to be a necessary element for development of obstructive hydrocephalus, the presence of a communicating form suggests that the ependymal layer is involved in CSF distribution and reabsorption in different ways. It not only preserves patency of the cerebral ventricles and aqueducts, but also allows the passage of fluids and solutes between the brain and CSF spaces.

Given the observation that the underlying glia attempts to re-form a layer similar to the native ependyma, one would think that physical ependymal disruption alters the balance of fluids and solutes across this interface. We hypothesize that three separate mechanisms are implicated in this form of hydrocephalus in mouse models and humans: (1) cerebral aqueduct and subarachnoid space obstruction caused by ependymal that takes place around day 12 of embryonic life might impair CSF movement *via* the paravascular route and dural granulations; (2) abnormal function or absence of multiciliated ependymocytes might curtail the flow of CSF along the ventricles, aqueduct, subarachnoid spaces; (3) given the contribution of the ependyma to fluid transfer with the brain interstitium and its evolution during embryonal life, we hypothesize that ependymal loss may result in abnormal movement of CSF and water from the brain parenchyma to the cerebral ventricles.

It is known that embryonal CSF has a significant concentration of proteins and solutes, and that ependymal tight junctions are expressed at high levels. Any damage to the neuroependyma could therefore result in interstitial fluid transfer toward the ventricles by means of osmosis, explaining certain forms of transient communicating hydrocephalus seen in models of embryonal junctional derangement.

**Figure 3:**
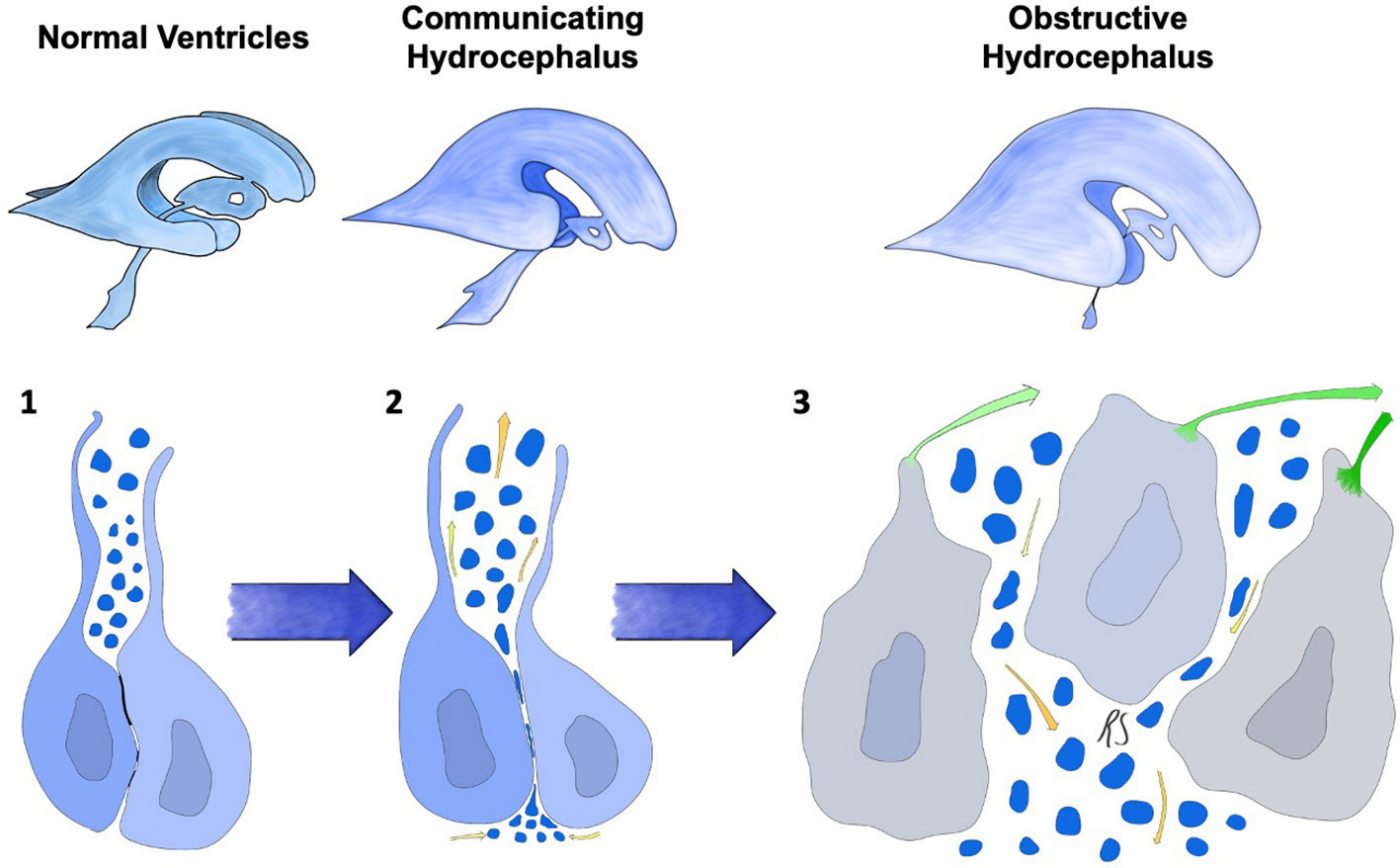
Progression of ependymal failure and development of hydrocephalus. (1) normal ependyma with intact junctions, solute/CSF interchange is maintained. (2) junctional damage with increased transependymal flow toward the ventricles represented by yellow arrows and water particles penetrating into the ependymal layer. This could be caused by an osmotic gradient secondary to high CSF protein concentration. A form of communicating hydrocephalus may develop at this stage. (3) failure and collapse of the ependymal layer, with denudation of the ventricular wall and aqueductal/subarachnoid occlusion. This mechanism could lead to obstructive hydrocephalus. Green arrows represent ependymocytes detaching from the ependymal basement membrane and causing ventricular denudation. Trans-ependymal flow of water and solutes is at this point uncontrolled, leading to additional ventricular enlargement, further ependymal distension and damage.

In conclusion, (Figure 3, Stage 2) in these models a two-stage mechanism could be at the basis of the switch from communicating to obstructive hydrocephalus. The first form ensues when osmotic centripetal movement of fluids from the interstitium takes place, while obstructive hydrocephalus is a mere consequence of the final collapse of ependymal walls with obstruction of the cerebral aqueduct and Pacchioni's granulations (Figure 3, Stage 3).

## 4. Conclusions

The ependymal lining, one of the main interfaces between the brain and CSF systems, has long been considered an inert and permeable layer of epithelial cells with almost no barrier function. However, recent studies suggested that this interface undergoes several changes during *in utero* and postnatal life, mainly secondary to variable expression of different paracellular junctional proteins. By harnessing the characteristics of these complexes, it may be possible to understand several forms of congenital communicating and obstructive hydrocephalus. Further, differential expression of junctional proteins could lead to selective modulation of the ependymal permeability, allowing for its use as an avenue for CSF redistribution, as well as a local site of drug administration.

## Data availability statement

The original contributions presented in the study are included in the article/supplementary material, further inquiries can be directed to the corresponding author.

## Author contributions

RS and JS designed the study together. RS wrote the first draft of the manuscript and prepared the figures. JS contributed to and edited the final draft. Both authors approved the manuscript.
